# Site-Control of InAs/GaAs Quantum Dots with Indium-Assisted Deoxidation

**DOI:** 10.3390/ma9030208

**Published:** 2016-03-18

**Authors:** Sajid Hussain, Alessandro Pozzato, Massimo Tormen, Valentina Zannier, Giorgio Biasiol

**Affiliations:** 1 IOM CNR, Laboratorio TASC, Area Science Park Basovizza, S.S. 14 Km 163.5, 34149 Trieste, Italy; mshussain76@gmail.com (S.H.); pozzato@iom.cnr.it (A.P.); tormen@iom.cnr.it (M.T.); valentina.zannier@nano.cnr.it (V.Z.); 2 Department of Physics, University of Trieste, Via Valerio 2, 34128 Trieste, Italy

**Keywords:** molecular beam epitaxy, quantum dots, nanostructures, nanoimprint lithography, self-assembly, compound semiconductors, GaAs, InAs, 81.07.Ta, 81.15.Hi, 68.35.bg, 81.16.Nd

## Abstract

Site-controlled epitaxial growth of InAs quantum dots on GaAs substrates patterned with periodic nanohole arrays relies on the deterministic nucleation of dots into the holes. In the ideal situation, each hole should be occupied exactly by one single dot, with no nucleation onto planar areas. However, the single-dot occupancy per hole is often made difficult by the fact that lithographically-defined holes are generally much larger than the dots, thus providing several nucleation sites per hole. In addition, deposition of a thin GaAs buffer before the dots tends to further widen the holes in the [110] direction. We have explored a method of native surface oxide removal by using indium beams, which effectively prevents hole elongation along [110] and greatly helps single-dot occupancy per hole. Furthermore, as compared to Ga-assisted deoxidation, In-assisted deoxidation is efficient in completely removing surface contaminants, and any excess In can be easily re-desorbed thermally, thus leaving a clean, smooth GaAs surface. Low temperature photoluminescence showed that inhomogeneous broadening is substantially reduced for QDs grown on In-deoxidized patterns, with respect to planar self-assembled dots.

## 1. Introduction

The use of self-assembled quantum dots (QDs) has been suggested as a promising approach in applications as building blocks in single-photon sources and novel quantum computer architectures [1,2]. Such applications would greatly benefit from a spectral and spatial control of QDs [3]. In this regard, combined top-down and bottom-up techniques have been developed, in which self-assembly of epitaxially grown dots is directed onto regular arrays of pre-patterned holes [4]. In the most studied system, site-controlled InAs/GaAs QDs grown by Molecular Beam Epitaxy (MBE), a critical step is the deoxidation process of the patterned substrate: in order to preserve the hole arrays and to achieve a high surface flatness, thermal deoxidation is preferably replaced by a controlled exposure of the surface to atomic beams at lower temperature, which transforms surface oxides into volatile species. Such alternative procedures must also effectively remove surface contaminants, since only thin GaAs buffer layers can be grown before the QD arrays, to avoid planarization of the hole profile. Among the atomic species, hydrogen has been the most widespread one, as it leads to a clean, low-roughness surface with well-preserved hole shapes [5]. As hydrogen sources may not be available or compatible with all MBE systems, and would anyway require a dedicated apparatus, Ga beams have been used as an alternative [6,7]. The method has proven effective in removing oxides from patterned GaAs surfaces [8], however it has two drawbacks. First, it requires a careful optimization of the Ga dose, since any excess material deposited beyond complete oxide removal results in metallic droplets forming on the de-oxidized surface. Due to the low Ga vapor pressure, such excess metallic Ga would re-evaporate significantly only at temperatures higher than 650C [9]. Thus, extreme care is required in depositing the Ga critical dose required to completely remove the oxides, but avoid droplet formation [7]. Second, it leads to a substantial hole filling and elongation in the [110] direction due to anisotropic Ga diffusion, favoring the formation of multiple dots per hole [8]. It would thus be desirable to find an alternative deoxidation method, which provides the same surface quality and pattern preservation of atomic hydrogen, but with the technological simplicity of gallium.

In this work, we have used surface exposure of nanopatterned GaAs surfaces to indium beams as a valid alternative to Ga and H, since In sources are a standard component of III/V MBE systems, and excess In can be easily removed thermally at low enough temperatures (about 550C), due to its higher vapor pressure with respect to Ga [9,10]. This deoxidation technique has allowed the formation of high quality InAs QDs on planar GaAs surfaces, exhibiting sharp single-dot emission lines [11]. We have shown by Reflection High Energy Electron Diffraction (RHEED) and X-ray Photoemission Spectroscopy (XPS) that surface oxides were completely removed by In-assisted deoxidation (IAD), and that any excess indium could be completely re-evaporated on clean planar surfaces, for optimized procedures. We have then compared the hole shape and QD regrowth on nanopatterned GaAs arrays cleaned either by Ga-assisted deoxidation (GAD) or IAD. Most importantly, we have found that In-assisted oxide desorption, besides easily providing clean, monolayer smooth surfaces, has the additional beneficial effect to hinder hole elongation in the [110] direction upon subsequent deposition of thin GaAs buffers. Overgrowth of InAs QD stacks has shown that single-dot occupancy per hole is greatly favored, with respect to identical structures grown on patterns cleaned by GAD. Finally, the optical properties of single InAs QD layers grown on multiple In_0.5_Ga_0.5_As QD stacks on IAD patterns were assessed by low-temperature Photoluminescence (PL) spectroscopy.

## 2. Materials and Methods

### 2.1. Pre-Growth of Buffer Layers

Substrates for synthesis of site-controlled QDs were pre-grown by MBE on semi-insulating (001) GaAs substrates on which 200 nm GaAs, 200 nm GaAs/Al_0.33_Ga_0.67_As superlattice (SL), and further 200 nm GaAs were deposited as a buffer to improve surface quality. Growth is performed in a Gen II solid-source MBE system (Veeco Instruments Inc, St. Paul, MN, USA), with growth rates of 1 µm/h for GaAs and 0.5 µm/h for AlAs, a V/III ratio of about 20 for GaAs and a growth temperature of 580 °C.

### 2.2. Nanofabrication of Hole Patterns

**Figure 1:**
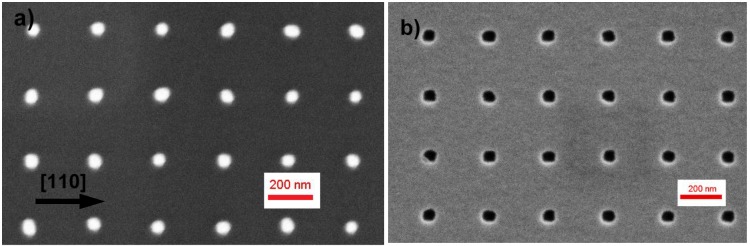
SEM images of (**a**) the silicon mold used for NIL patterning of GaAs; (**b**) a patterned GaAs (001) surface.

Samples were then patterned with square arrays of nanosized holes on a quarter of 2” wafer by conventional Nanoimprint Lithography (NIL). Among the patterning techniques, NIL has been recently employed thanks to its high throughput and low cost for manufacturing of miniature devices [12]. This technique has in fact the capability to scale up patterns, which can fabricated by electron beam lithography on at most some mm^2^ areas for research purposes, to regions of several cm^2^, which would be required for broad-area optoelectronic devices based on site-controlled nanostructures. A Si mold with a 2D square array of cylindrical pillars of about 90 nm height, 64 ± 6 nm diameter and a period of 300 nm, fabricated in our lab (Figure 1a), was used to transfer a hole pattern on a resist layer (mr-I 7010E, microresist GmbH) directly spun on GaAs, on surfaces up to a quarter of a 2” substrate. In the NIL process, a pressure of 7.5 MPa was applied at 90 °C for 8 min. An oxygen plasma treatment ensured complete removal of residual resist at the hole location.

**Figure 2:**
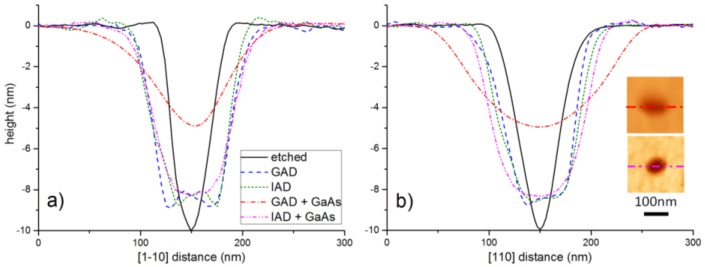
AFM line scans of hole profiles along (**a**) [1$\bar{1}$0] and (**b**) [110] after wet chemical etching, GAD, IAD, GAD followed by 10 nm GaAs, and IAD followed by 10 nm GaAs (each profile represents an average over 15–20 line scans). Inset of panel (b): AFM images of two holes after GAD and IAD, followed by 10 nm GaAs in both cases.

The pattern was then transferred to the GaAs substrate by wet chemical etching in a 1:1:25 H_3_PO_4_:H_2_O_2_:H_2_O solution. Resist residuals were removed by solvent baths and oxygen plasma cleaning. Before introduction into the MBE chamber, patterned samples were dipped in a HCl:H_2_O solution (1:5) for 30s to thin down the surface oxide layer. Figure 1b shows a Scanning Electron Microscopy (SEM) image of the patterned surface before introduction into the MBE chamber. A highly uniform hole array was obtained, with nearly circular holes with approximate lateral diameters of 55 and 50 nm in the [110] and [1$\bar{1}$0] directions, respectively, and a dispersion as small as 2 nm. Interestingly, nanoholes resulted to be smaller and more uniform than the pillars on the mold, probably due to the smaller diameter of the pillar at the tip, which is not easy to imagine by SEM. The hole depth measured in Atomic Force Microscopy (AFM) (Autoprobe CP, ThermoMicroscopes, Sunnyvale, CA, USA) was about 10 ± 0.5 nm. AFM of line scans along [1$\bar{1}$0] and [110] are shown as black traces in Figure 2.

### 2.3. Oxide Removal Procedures and Epitaxial Growth of Nanostructures

**Figure 3:**
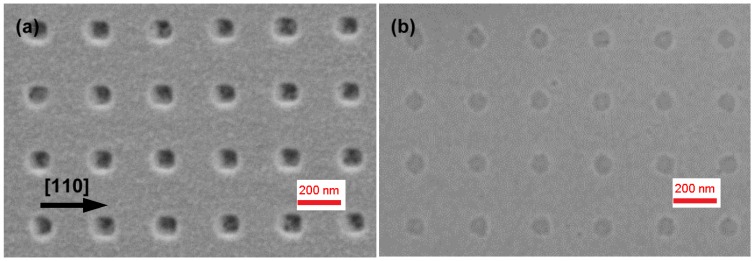
SEM images of a patterned GaAs surface after *in situ* oxide removal by (**a**) Ga-assisted deoxidation; (**b**) In-assisted deoxidation.

In the MBE chamber, we have removed the native surface oxide by exposure either to Ga or In beams. For GAD, a series of Ga pulses with equivalent GaAs thickness of 0.2 nm and growth rate of 0.067 nm/s was alternated with 15 s growth interruptions at 450 °C, similarly to the procedure of Wasilewski *et al.* [6]. The optimal Ga dose was defined by monitoring the intensity of the specular RHEED spot as an indication of surface crystallinity and flatness [6]. As the oxide is thinned down by Ga exposure, specular intensity during growth interruptions increases after each cycle, until all the oxide is removed. After 5 Ga cycles, intensity saturates as a signature of a complete oxide removal. Further Ga deposition leads to a decrease of the specular RHEED spot, due to the formation of metallic Ga droplets. We have thus identified an optimal Ga exposure of 5 cycles (corresponding to an equivalent GaAs thickness of 1 nm) for oxide removal. In-assisted deoxidation (IAD) was carried out at 500 °C, with an equivalent InAs growth rate of 0.04 Å/s. The surface was exposed to a continuous In flux until the onset of a (4 × 1) RHEED pattern was visible (corresponding to about 1.5 nm equivalent InAs thickness), indicating the presence of metallic In on the surface. Note that In-rich InAs/GaAs (001) surfaces are generally indicated by a (4 × 2) reconstruction [13], however the 2× may not be always clear [14], likely depending on the stoichiometry, and IAD of planar GaAs (001) gave (4 × 1) RHEED patterns similar to ours [10]. The samples were then in both cases annealed to 560 °C, leading to a sharp (1 × 1) reconstruction. In the case of IAD, the disappearance of the (4 × 1) pattern indicated the desorption of excess In from the surface [10]. Once the temperature is reached, samples were then exposed to As_4_ flux for 2 min leading in both cases to a sharp (2 × 4) RHEED pattern. On planar test samples, both In- and Ga- assisted treatments yielded sub-ML flat surfaces (RMS roughness ≈ 0.2 nm, comparable or slightly better than similarly treated surfaces [6,10]) without visible pits or droplets. On the patterned samples, in both cases the holes widened by about 50% and largely maintained their symmetrical shape, reaching an average lateral size of about 80–85 nm, with a slight depth reduction to about 9 nm. Figure 3a,b shows SEM images of the Ga- and In-deoxidized patterns, respectively, while AFM line scans are shown in Figure 2 (blue and green traces, respectively).

Subsequent GaAs growth was performed at 460 °C with a growth rate of 1.7 Å/s and a V/III ratio of 20. For all the samples, InAs QDs were obtained by growing 1.8 ML of InAs at the growth temperature of 500 °C, with pulsed deposition (0.1 ML/cycle), a growth rate of 0.1 ML/s and a V/III pressure ratio of about 200.

### 2.4. Chemical and Optical Characterization

Chemical analysis of the deoxidized and grown surfaces were performed *in situ* by XPS (SSX-100, Surface Science Laboratories, Inc., Mountain View, CA, USA). Al kα radiation was used for XPS measurements, corresponding to a photoelectron mean free path of 1.5 nm for our geometry.

The optical properties of site-controlled QDs were assessed by means of low-temperature PL (13.5 K), by using a 514.5 nm Ar^+^ laser, with a power of 5 mW.

## 3. Results and Discussion

### 3.1. Chemical Analysis of Oxide Removal

**Figure 4:**
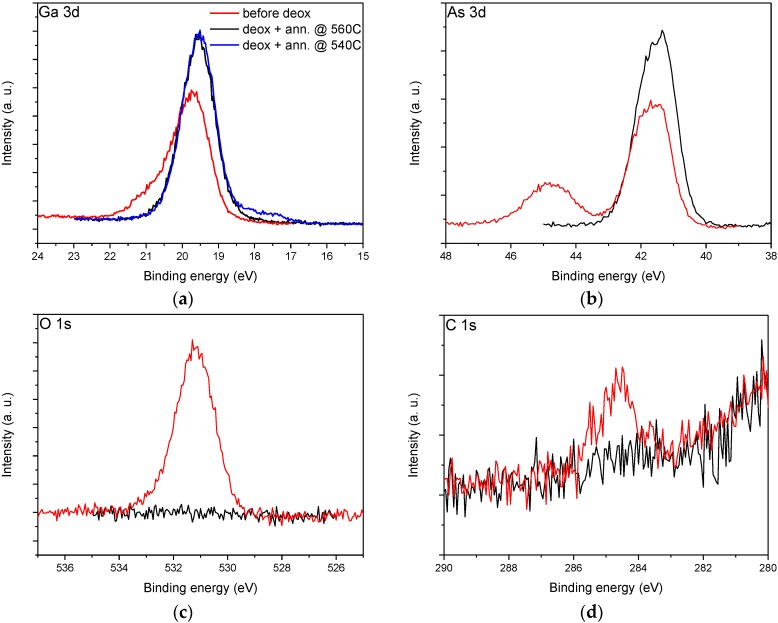
XPS spectra of (**a**) Ga 3d; (**b**) As 3d; (**c**) O 1s and (**d**) C 1s core level emissions before oxide removal (red traces) and after In-assisted oxide removal and annealing at 560 °C (black traces) and 540 °C (blue trace, Ga 3d only).

To assess the chemical composition of In-deoxidized surfaces, we have performed XPS experiments on planar, n-doped GaAs (001) wafers, pre-treated before insertion into the MBE and deoxidized as described above. Photoemission peaks from the Ga 3d, As 3d, O 1s and C 1s core levels before (red traces) and after deoxidation and annealing at 560 °C (black traces) are shown in Figure 4. Spectra from the as-introduced sample show clear signs of oxidation. Emission related to Ga_2_O_3_ can be seen in the Ga 3d peak at 20.8 eV, separated by 1.3 eV from the bulk GaAs component. The As 3d emission shows an AsO_x_ component at 44.5 eV (As 3d 5/2), separated by 3.1 eV from the bulk GaAs, whose oxidation state could not be resolved with our energy resolution. In addition, a clear O 1s emission was present. After annealing to 560 °C, the photoemission spectra indicate a complete removal of oxygen from the GaAs surface, as evidenced by the disappearance of the O 1s signal and of the oxide-related components in the Ga 3d and As 3d core levels. Regarding carbon contamination, pre-growth plasma and HCl treatment was able to remove most of it, by leaving only a barely visible C 1s trace above the noise level. In-assisted deoxidation removed the remaining traces on the surface below the detection limit of our XPS setup. Note that a similar surface chemistry, with complete removal of oxidized Ga and As species, and a reduction of C 1s signal virtually down to the noise level, was obtained by hydrogen exposure [15]. For Ga 3d emission, we show also a spectrum obtained after annealing at 540 °C with the rest of the process unchanged (blue trace). In this case, a small shoulder at lower binding energies is visible, due to In 4d emission, and indicating that at this temperature a small residual excess In is still present on the surface. All the other photoemission peaks are identical at 540 (not shown) and 560 °C.

### 3.2. Deposition of GaAs Buffer Layers

**Figure 5:**
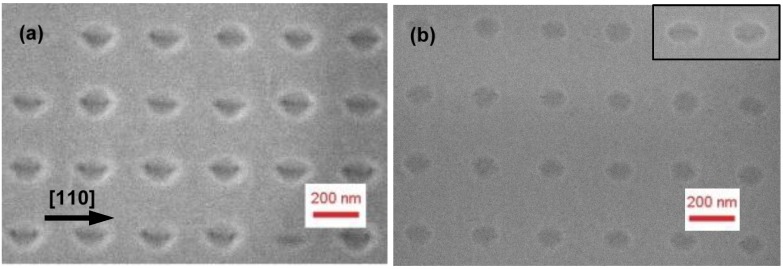
SEM images of 10nm GaAs deposited on a Ga- (**a**) and In-deoxidized (**b**) patterned GaAs surface. Inset in (b) shows two holes of an IAD pattern annealed for longer times (10 min) after In deposition.

Patterned samples were then overgrown with a 10 nm GaAs buffer at 460 °C, leading on both cases to a sharp (2 × 4) RHEED pattern. Figure 5a,b shows SEM images of the hole arrays in the case of GAD and IAD, respectively. It can be clearly seen that for GAD the holes are considerably elongated in the [110] direction, reaching an average size of about 135 × 80 nm^2^. This elongation is similar to what has been observed for GaAs overgrowth on holes deoxidized by hydrogen exposure [16], as a result of faster incorporation rate of GaAs on As-terminated B-type facets lying parallel to the [110] direction [17]. In the case of IAD, however, the holes preserve much better their shape upon growth of 10 nm GaAs and result to be much more symmetric, with average size 100 × 80 nm^2^. We speculate that the better preservation of the hole shape in the [110] direction could be due to a small residual presence of InAs in the holes, which would hinder successive GaAs nucleation inside the holes themselves, due to strain induced by InAs. This mechanism can be formalized in terms of the chemical potential on a non-uniform surface profile, which for our growth environment can be written as $µ\left(r\right)={µ}_{o}+\frac{Ω}{2E}\sigma_{\tau }{\left(r\right)}^{2}+\gamma Ω\kappa \left(r\right)$ [18]. Here, µ_o_ is the chemical potential on a flat, uniform surface; the second term is the chemical potential variation due to non-uniform surface strain, with σ_τ_ the tangential stress and *E* the elastic modulus; the third term is the surface energy contribution to the chemical potential, where γ is the surface free energy, Ω the atomic volume and κ(r) the surface curvature, and reflects the tendency of adatoms to migrate towards concave regions (capillarity). During In-assisted oxide desorption, In atoms diffuse preferentially into the holes due to capillarity. This In accumulation could prevent a complete material desorption inside the holes during annealing, differing from the planar areas, and results in the formation of a thin InAs layer upon exposure to arsenic. The strain induced by this InAs would then hinder GaAs nucleation in the holes, due to the second term of the equation above, and thus help to preserve the hole shape, which would be altered by asymmetric incorporation of GaAs. This explanation is confirmed by a test sample in which an IAD pattern was annealed for much longer times (10 min) in As_4_ flux before depositing the GaAs buffer, with the intent to desorb any possible residual In inside the holes. In such sample (upper right inset in Figure 5b) the holes tend to elongate similarly to the GAD case (about 140 × 80 nm^2^), suggesting that the different hole shapes between the GAD and IAD procedures is actually due to residual In in the holes, which gets desorbed with longer annealing. The reduced deposition of GaAs into the holes, in the case of IAD, is confirmed by their larger depth after deposition of 10nm GaAs, with respect to GAD. Figure 2 shows AFM line scans along both directions for the two cases: while for GAD (red traces) the hole depth is reduced to about 5 nm, for IAD (purple traces) it is about 8.5 nm, with just a 1.5 nm reduction with respect to the etched holes. Such scans allow to better appreciate the superior preservation of the hole shape in the case of IAD, with substantially no alteration along [1$\bar{1}$0] and only a slight enlargement along [110], after GaAs deposition.

### 3.3. Growth of InAs Quantum Dots

The hole shape has a deep influence on the spatial distribution of overgrown InAs QDs. We have deposited on both patterns either a single layer of InAs (1.8 ML thickness), or a tenfold stack of 1.8 ML InAs separated by 10 nm GaAs spacers. Such spacer thickness was chosen as the highest which would guarantee a 95% vertical pairing probability between dot stacks through strain propagation [19].

**Figure 6:**
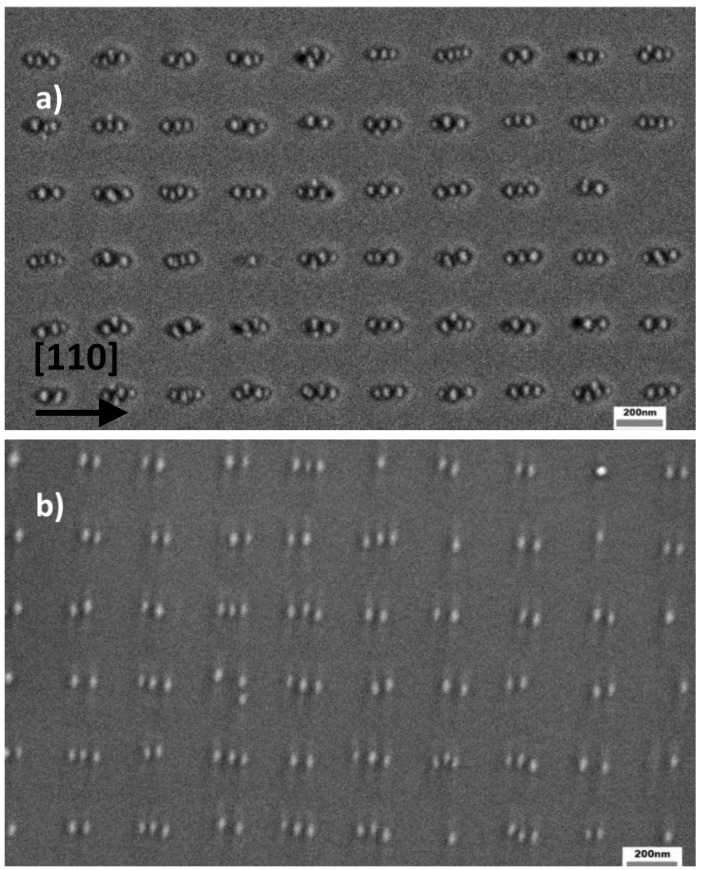

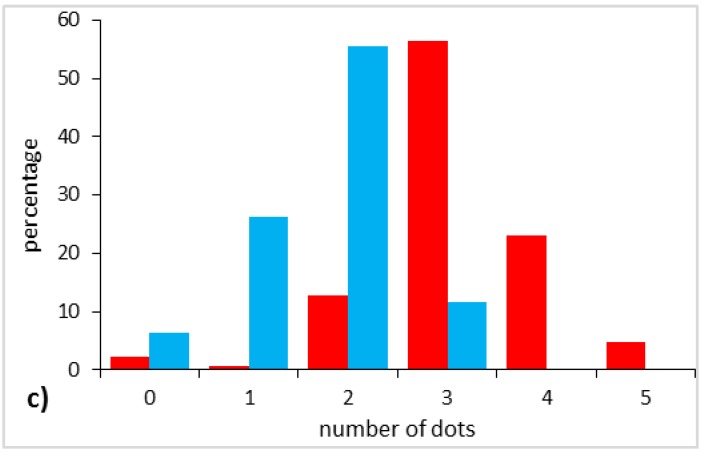
SEM images of (**a**) a single layer and (**b**) a tenfold stack of site-controlled InAs/GaAs QDs grown on a nanopatterned GaAs hole array, deoxidized by Ga beams; (**c**) histogram showing the dot occupancy per hole on GAD patterns for a single layer (red) and a tenfold stack of InAs QDs (blue).

In the case of GAD, due to the much larger size of the holes in the [110] direction with respect to the lateral dot dimensions (around 30nm), one hole provides nucleation sites to accommodate several dots in such orientation. Due to the difficulty of depositing exactly the InAs amount giving the correct equivalent planar density corresponding to the density of the holes [20], this resulted in the actual formation of multiple dots per hole, as shown in Figure 6a. Notice that even the in few sites with single dots (one is visible near the center of the figure), the QDs do not form at the hole center but rather on its sides, likely due to the presence of a high density of reactive steps. A similar behavior was observed for InAs dots grown on nanopatterned GaAs pyramids, where they tend to nucleate on high-index, highly reactive lateral facets [21]. As can be seen in the histogram of Figure 6c, more than 80% of the holes are occupied by three or more dots, while single-dot occupancy is negligible. The average dot diameter was 28 ± 12 nm, where the large non-uniformity could be due to the widely inhomogeneous spatial distribution of the dots in each hole. When stacks of QDs are grown, the dot distribution is basically reproduced for the successive layers, as vertical strain fields force dot in the growing layers to form atop of the buried ones [19]. This is seen in Figure 6b, which shows a tenfold stack of InAs QDs. A histogram of the dot occupancy is shown in Figure 6c, where it can be seen that about 70% of the holes contain double or triple dots, with only a slight reduction of the occupancy number with respect to the first layer. Thus, growth of multiple stacks does not reduce significantly the hole occupancy as the strain fields of the dots remain laterally separated, and give rise to multiple dots in each hole site similarly to the first layer [22]. A reduction in the InAs amount in each layer simply reduced the dot size without affecting their number. In this case too it can be noted that the few single dots are not aligned along [1$\bar{1}$0] from hole to hole (see the sites on the right side of Figure 6b), as they nucleate atop either side of the underlying holes.

**Figure 7:**
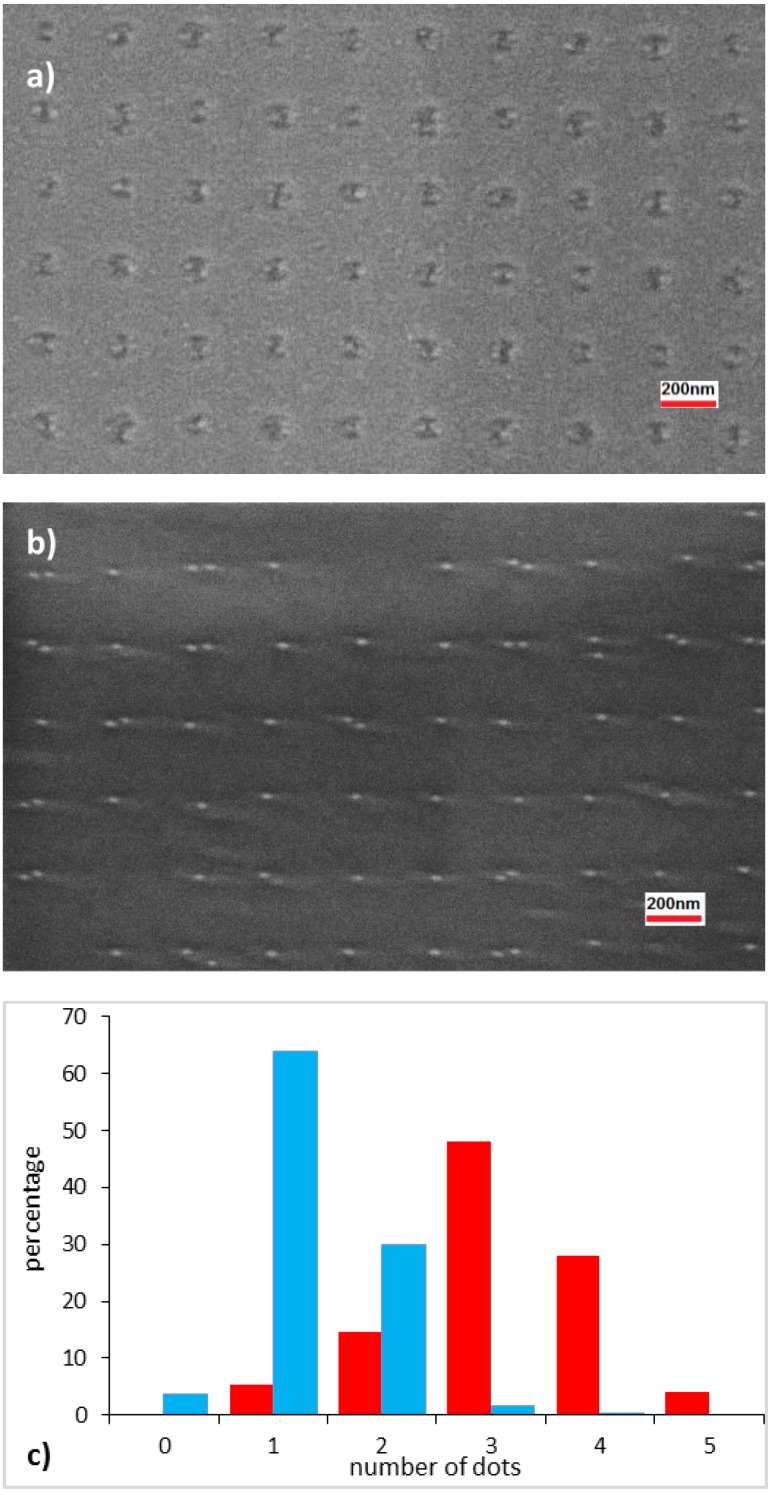
SEM images of (**a**) a single layer; and (**b**) a tenfold stack of site-controlled InAs/GaAs QDs grown on a nanopatterned GaAs hole array, deoxidized by In beams; (**c**) histogram showing the dot occupancy per hole on IAD patterns for a single layer (red) and a tenfold stack of InAs QDs (blue).

**Figure 8:**
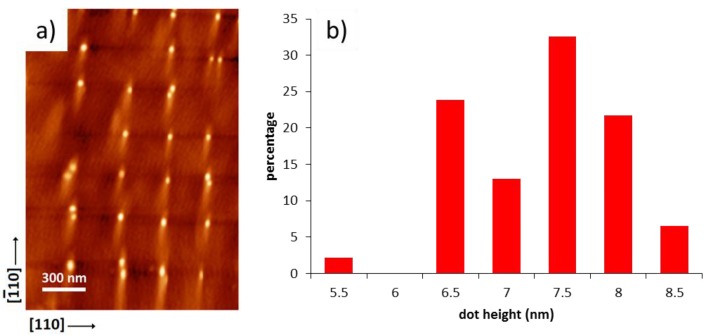
(**a**) AFM image of a tenfold stack of site-controlled InAs QDs grown on In-deoxidized hole arrays; (**b**) histogram of the height distribution relative to *single* dot-per-hole only.

For QDs grown on patterns deoxidized by In exposure, multiple dots are still formed in each hole (Figure 7a). However, they are more closely spaced and symmetrically distributed than in the previous case, due to the much more rounded hole shape, which is not altered during InAs deposition. The statistics of occupancy (Figure 7c) are very similar to those of the equivalent deposition of the first QD layer in the case of GAD (most of the holes contain 3 dots). The average dot diameter was 22 ± 6 nm, *i.e*., slightly smaller and more uniform than in the equivalent GAD case; this could be possibly due to a more closely packed and uniform arrangement inside the holes. Despite of the initial multiple occupancy, the spatial distribution of the first QD layer favors the nucleation of single dots in the subsequent layers. This can be seen in the SEM image of Figure 7b, relative to a tenfold QD stack. The distribution of occupancy (Figure 7c) shows that about 64% of hole locations accommodate a single dot, thus with a substantial improvement over the same structure grown on GAD patterns. The percentage of single-dot occupancy could be even higher than this, since part of the void locations are likely due to a missing hole, and thus be ascribed to fabrication faults, rather than to epitaxy. The average size of single dots resulted to be much larger and more uniform than in the first stack (42 ± 4 nm diameter), since they do not have to compete with neighboring dots for the SK growth, and they feel a much more uniform environment. Note that the (few) single dots in the 10th layer of the GAD case have a similar size distribution (44 ± 6 nm). The height distribution of the 10th QD layer for the IAD case was measured by AFM (see Figure 8). The average height, relative to *single* dots-per-hole only, was found to be 7.1 ± 0.6 nm, corresponding to an 8.5% dispersion. The single dot uniformity is thus very close to the record height uniformity of ±7.2% reported by Kiravittaya *et al.* for optimized single dots on patterns defined by electron beam lithography (EBL) [20] (no statistics on uniformity were reported so far for NIL-defined site-controlled dots). These results show that, by careful optimization of the fabrication and growth processes, it is possible to extend site-controlled self-assembled QDs from small, EBL-defined patterns to large area arrays, by maintaining a similar quality for what concerns their structural uniformity.

Since the growth parameters and QD density in the first layer (about 3.1 dots per hole) are the same for the two deoxidation procedures, it is evident that the preponderant establishment of single dots per hole in the case of IAD is due to the different arrangement of the dots inside the holes in the first layer. In particular, such different arrangement should originate a different evolution of the strain fields above the dots in the two cases. Namely, the more symmetrical hole shape induced by IAD, should favor an intermixing of the strain fields of neighboring dots, which eventually join to form more localized single strain minima atop of most of the holes. An asymmetry in the strain energy density distribution was evidenced by calculations performed on QD bimolecules aligned either along [110] or [1$\bar{1}$0] [22]. Consistently with our findings, strain fields above double QD aligned along [110] were found to remain separated, giving origin to multiple dots with the same spatial distribution in the subsequent layers. If, however, two neighboring dots are aligned along [1$\bar{1}$0], their strain fields tend to merge, thus providing a *single* strain minimum (and hence a single nucleation site) for dots in the following layers. Since all dots in the GAD case are aligned along [110], their strain fields propagate separately through successive layers, and QD distribution mimics the one of the initial seed layer. In the IAD case, QD distribution in the round holes is largely symmetrical, thus a considerable part of neighboring dots will merge their strain fields and evolve into single dots in the following layers.

### 3.4. Optical Characterization

**Figure 9:**
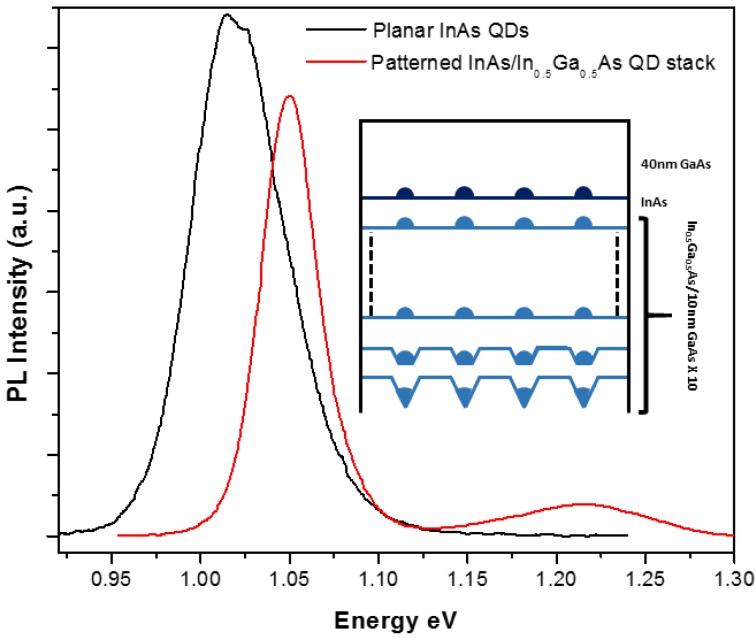
Low T PL spectra of single layer of InAs QDs grown on a planar substrate (black line), and of a 10X In_0.5_Ga_0.5_As + single InAs QD stack grown on patterned substrate (red line). Inset shows a schematic drawing of the growth sequence for the latter sample.

We have studied the optical properties of InAs dots grown on IAD patterns, on a sample containing a tenfold stack of In_0.5_Ga_0.5_As QDs, followed by a single layer of InAs QDs and a GaAs cap of 40 nm (see inset of Figure 9). By replacing InAs with In_0.5_Ga_0.5_As in the underlying stacks, emission from the topmost InAs QD layer is well separated spectrally from the underlying dots. In this way, it is possible to observe the optical properties of a single QD layer, and to avoid effects of inhomogeneous broadening due to multiple dots per hole in the first stacks (we have verified, in a sample grown like in the inset of Figure 9 but without GaAs cap, that the dot distribution of the last layer is the same as in Figure 7b,c)). In addition, the InAs QDs are kept well separated from the substrate, thus minimizing deleterious effects of residual surface contamination and defects on the optical emission.

Low temperature PL emission from this sample is shown in Figure 9 (red trace). The InAs QDs emit at 1.051 eV with a FWHM of 29.6 meV; this peak is well separated from a higher energy, much broader (about 70 meV FWHM) and less intense emission, which could be ascribed to In_0.5_Ga_0.5_As QDs. Similar spectra were obtained on a comparable sample in Reference [23], where RT photoluminescence emission was shown. The linewidth that we have measured compares well with results obtained by other groups, although narrower emission was obtained from dot arrays grown on EBL-defined patterns, likely due to a more accurate optimization of the 1-to-1 dot occupancy in the holes [20]. In fact, our height statistics relative to *single* dot-per-hole alone are very similar to those of that work (see above), thus we expect that a good part of the inhomogeneous broadening we observe is related to the residual presence of multiple dots in part of the holes. Regarding site-controlled QDs grown on NIL-defined hole patterns, slightly larger linewidths have been reported in Reference [24], where however a single InAs QD layer was grown at only 5 nm from the etched surface. We have compared the optical emission of our sample with that of a single InAs QD layer with comparable density, grown on a planar surface with a thick buffer layer (200 nm GaAs, 200 nm GaAs/AlGaAs SL, 200 nm GaAs), thus in the optimal conditions not to feel the influence if impurities from the substrate. Such spectrum is shown as well in Figure 9 (black trace). Similarly to Ref. [23], the slightly larger average size of planar dots resulted in a redshifted optical emission, with respect to the patterned ones. More importantly, emission from dots grown on the patterned substrate is about twice as sharp, due to the higher size uniformity. Besides, the integrated intensity is comparable to that of the planar QD, showing that our procedures for substrate preparation, cleaning and deoxidation were able to minimize the deleterious effects of surface-related defects and impurities on our site-controlled QDs.

## 4. Conclusions

We have shown that In-assisted deoxidation is an effective method to yield clean and smooth GaAs surfaces, in accordance with previous observations [11]. Furthermore, if applied on surfaces patterned with regular arrays of holes, this method greatly helps to preserve the original hole shape after deposition of a GaAs buffer, in contrast to alternative procedures [16]. This shape preservation could be ascribed to a combined effect of capillarity and strain on Ga diffusion, induced by the possible presence of residual InAs in the holes. Upon overgrowth of single InAs QDs layers on the patterned arrays, the more rounded shape of the holes in case of IAD, as compared to GAD, favors a more uniform distribution of multiple dots in the holes. As a consequence, this uniform distribution helps to reach a better single-dot occupancy per hole when multiple QD stacks are grown, through a more efficient intermixing of the strain fields of the dots from the first stacks. Finally, QDs grown in IAD patterns exhibit a much sharper photoluminescence emission, as compared to planar self-assembled dots, without significant reductions of the signal intensity.
